# The use of thyroid cartilage needle electrodes during intra-operative nerve monitoring in thyroid surgery: A multi-center retrospective study

**DOI:** 10.1007/s13304-025-02178-1

**Published:** 2025-04-23

**Authors:** Kaan Balcı, Yiğit Türk, Murat Özdemir, Paulina Kuczma, Christophe Tresallet, Che-Wei Wu, Tzu-Yen Huang, Adi Syazni Muhammed, Rohaizak Muhammad, Shahrun Niza Bin Abdullah Suhaimi, Nani Harlina, Matija Buzejic, Vladan Zivaljevic, Milan Jovanovic, Özer Makay

**Affiliations:** 1 Department of General Surgery, Division of Endocrine Surgery, Ege University Hospital, Izmir, Turkey; 2 Department of Digestive, Bariatric and Endocrine Surgery, Avicenne University Hospital, Sorbonne Paris Nord University, Assistance Publique-Hôpitaux de Paris, Bobigny, France; 3 Department of Otolaryngology, Kaohsiung Medical University Hospital, Kaohsiung, Taiwan; 4 Breast & Endocrine Surgery Unit, Department of Surgery, Hospital Canselor Tuanku Muhriz UKM, Kuala Lumpur, Malaysia; 5 Clinic for Endocrine Surgery, University Clinical Center of Serbia, Belgrade, Serbia; 6 Center of Endocrine Surgery, Ozel Saglik Hospital, Izmir, Turkey; 7 Department of General Surgery, Aristotle University, School of Medicine, Thessaloniki, Greece

**Keywords:** Intraoperative nerve monitoring, Thyroid cartilage needle electrodes, Loss of signal, Adverse event, Recurrent laryngeal nerve

## Abstract

In previous studies, the use of thyroid cartilage needle electrodes (TCN) was defined as an inexpensive method for intra-operative nerve monitoring (IONM) in thyroid surgery. This multi-center retrospective study aims to determine the effectiveness and reliability of TCN in thyroid surgery. Patients operated on between January 2018 and August 2023 from five centers were included in this study. Demographic data, indications, type of surgery, IONM recording system, pre–post-resection vagus nerve (V1–V2), pre–post-resection recurrent laryngeal nerve (R1-R2) amplitudes and latency values, type of loss of signal (LOS), adverse event (AE), intra-operative injury mechanism, and post-operative vocal cord examination (VCE) were evaluated. Patients with abnormal preoperative vocal cord examination were excluded. A total of 2105 patients (3772 nerves at risk) were included [1626 (77%) female, 479 (23%) male]; within this study, 1112 patients (53%) received a diagnosis of malignancy, while 993 (47%) were diagnosed with benign conditions. The mean initial vagus amplitude was 1093.74 µV (± 861.39). LOS occurred in 63 patients [Type 1 (84%), Type 2 (16%)] and AE in 36. No false-positive LOS occurred. Forty-six (87%) of LOS type 1 patients and nine (90%) of LOS type 2 patients had vocal cord palsy (VCP) during VCE (*p* < 0.05). In AE patients, there were only two (5.5%) patients who had vocal cord palsy during VCE (*p* < 0.05). VCP occurred in 57(2.7%) patients, with 9 (0.42%) remaining permanent. TCN is an inexpensive and feasible alternative to endotracheal tube electrodes and a system with satisfying amplitudes. It can also precisely predict post-operative vocal cord functions.

## Introduction

The recurrent laryngeal nerve (RLN) injury is the most significant complication to be avoided during thyroid surgery. Over the years, various methods have been employed to prevent RLN injury, with the recording of electromyography (EMG) signals in accordance with the guidelines of the International Neural Monitoring Study Group (INMSG) becoming a standard practice in thyroid surgery [1]. Although endotracheal tube (ETT) electrodes remain the most commonly utilized intra-operative nerve monitoring (IONM) technique in thyroid surgery, some of its disadvantages and high costs associated with ETT have driven the exploration of alternative IONM electrode applications. Despite the small sample sizes of these studies, they have served as a source of inspiration for our research [2–7].

The drawbacks of ETT electrodes include false loss of signal (LOS) due to tube malposition, tube rotation, saliva accumulation, lack of control by the surgeon, time delays caused by repositioning and tube manipulation, and the risk of inadequate contact with the vocal cords [2]. Studies by Turk et al. reported that the cost of IONM using thyroid cartilage needle (TCN) electrodes was approximately 20 times lower than with ETT electrodes [5], while Jung et al. found the cost to be 30 times lower [6].

Clinical and animal studies have reported that TCN electrodes are easy-to-use, convenient, result in minimal time loss, are under the surgeon’s control, and are safe [6–9]. TCN electrodes also offer additional technical advantages, including the ability to obtain high amplitude values, detect true LOS or adverse event (AE), inform surgical strategy, predict post-operative outcomes, and provide valuable assistance to the surgeon. According to INMSG guidelines, an initial amplitude of vagus nerve before resection (V1) > 500 µV or higher is recommended for a high-quality IONM evaluation.

In conclusion, our study demonstrates that TCN electrodes serve as a reliable and effective alternative to ETT electrodes in thyroid surgery. This is supported by IONM findings, such as higher initial amplitude and the absence of false-positive or false-negative LOS. Additionally, TCN electrodes offer ease of use and manipulation, making them a technically convenient option for nerve monitoring.

## Methods

The study included patients who were performed thyroidectomy with IONM via TCN from five different centers (Ege University Hospital, Department of General Surgery, Division of Endocrine Surgery, Turkey; Otolaryngology-Head and Neck Surgery at the Kaohsiung Medical University, Taiwan; Department of Digestive, Bariatric and Endocrine Surgery, Avicenne University Hospital, Sorbonne Paris Nord University, Assistance Publique-Hôpitaux de Paris, Bobigny, France; Clinic for Endocrine Surgery, University Clinical Center of Serbia, Belgrade, Serbia; Breast & Endocrine Surgery Unit, Department of Surgery, Hospital Canselor Tuanku Muhriz UKM, Kuala Lumpur, Malaysia) between January 2018 and August 2023. Ethical approval for the study was obtained from the ethics committees of all participating centers.

Each lead surgeon at the participating centers has at least 20 years of experience in thyroid surgery and has more than 350 cases per year.

TCN electrodes are preferred over conventionally used ETT electrodes due to several factors observed with ETT electrodes, including false-positive LOS caused by endotracheal tube malposition, tube rotation, saliva accumulation, lack of direct control by the surgeon as with needle electrodes, time-consuming tube repositioning and manipulation, insufficient vocal cord contact, and high cost.

The study assessed patient demographics; gender, surgical indications, type of surgery, IONM recording systems, pre–post-resection vagus nerve (V1–V2) amplitude and latency values, pre–post-resection recurrent laryngeal nerve (R1–R2) amplitude and latency values, types of LOS, AE, intra-operative recovery of LOS or AEs, mechanisms of intra-operative injury, post-operative vocal cord examination (VCE), and long-term VCE outcomes. Preoperative (L1) vocal cord examinations were conducted 24 h prior to surgery, and post-operative (L2) vocal cord examinations were performed for all patients as part of the study protocol.

### Inclusion & exclusion criteria

Patients aged over 18 years with a normal preoperative vocal cord examination were included in the study. Patients with abnormal preoperative vocal cord examinations, a history of neck radiotherapy, or tracheal or esophageal invasion were excluded.

### Technique of IONM via TCN

**Fig. 1:**
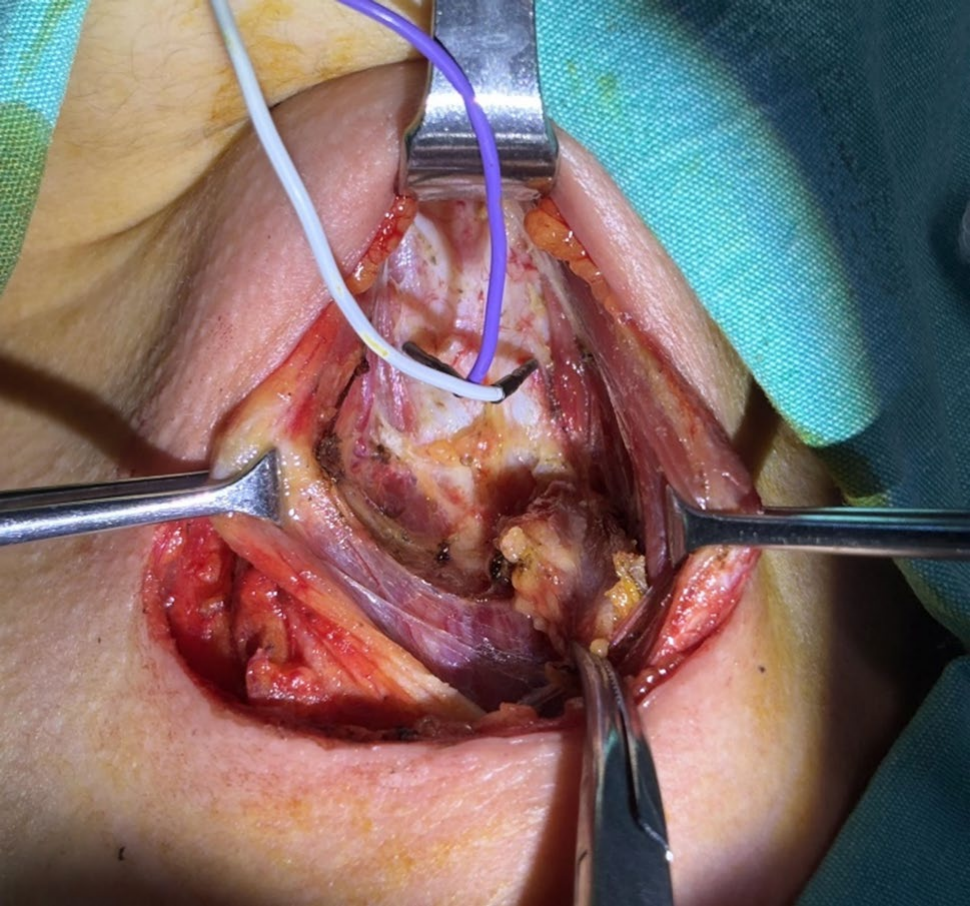
Final view of thyroid cartilage needle electrodes after insertion

The anesthesiologist administered 0.3 mg/kg of rocuronium bromide as a neuromuscular blocking (NMB) agent. Following the completion of pyramidal lobe dissection, the insertion site for the TCN electrodes was the lamina (perichondrium) of the thyroid cartilage on both sides, identified as region 6 in the study by Zhao et al. [3]. Each needle was inserted into region six on both sides as shown in Fig. 1*.*

The length of the needles used ranged from 13 to 17 mm and was composed of stainless steel. No significant differences were observed in the depth of insertion, and the current used ranged from 2 to 3 mA. The monitoring systems employed included Medtronic NIM™ 3.0 (Minneapolis, USA), Langer Avalanche monitor (Avalanche XD, Waldkirch, Germany), and Inomed C2 Xplore (Emmendingen, Germany).

### Measurements

In standard IONM, as recommended by the INMSG**,** amplitude values (measured in microvolts, µV) and latency values (measured in milliseconds, ms) are recorded as V1, R1, R2, and V2. These values are expressed for both sides of stimulation as mean ± standard deviation [1].

LOS is defined as an amplitude of 100 µV or less. In this study, we analyzed three distinct parameters within the LOS section:

1. LOS Type 1 (< 100 µV), distal RLN stimulation induces normal evoked activity. However, proximal stimulation to the injured segment elicits a waveform no greater than 100 μV [10, 11]
2. LOS Type 2 (< 100 µV), the exposed RLN shows no specific disruption site (i.e., diffuse or global RLN injury) and no visible change in appearance [10, 11].
3. AE, defined as a reduction of more than 50% from the initial amplitude but remaining above 100 µV [10].

LOS may result from various mechanisms associated with RLN injury during thyroid surgery. We categorized these mechanisms as unclear, traction, mechanical trauma, thermal, and transection.

According to the 2018 INMSG guidelines, intra-operative recovery of LOS is considered an important indicator for predicting VCP and determining the need for staged surgery [12].

Consequently, we evaluated three parameters for intra-operative recovery of LOS:

- No response.
- Recovery to > 100 µV but < 50% of the initial amplitude, further subdivided into two groups:
  - Type 1 LOS
  - Type 2 LOS.
- AE cases recovering to > 100 µV but < 50% of the initial amplitude (evaluated as negative), and recovery to > 50% of the initial amplitude.

VCP was assessed as two separate parameters: postoperative day 1 VCP, and Persistent VCP, defined as VCP persisting for 6 months or longer.

### Statistical analysis

Pearson correlation analysis was performed to compare LOS, AE, and mechanisms of injury, as well as the relationship between mechanisms of injury and intra-operative recovery of LOS and AE. The Chi-square test was used to evaluate whether VCP was observed on post-operative day 1 in patients with LOS or AE. The relationship between patients with LOS or AE who were evaluated as having VCP on post-operative day 1 and those who developed permanent VCP was assessed using Pearson correlation analysis. A *p* value of < 0.05 was considered statistically significant. Data analysis was conducted using IBM SPSS Statistics for Windows, Version 24.0 (IBM Corp., Armonk, NY, released 2016).

## Results

### Patients’ demographics

A total of 2105 patients underwent IONM using TCN electrodes, with 3772 nerves at risk. Of the patients, 1626 (77%) were female, and 479 (23%) were male. The surgical indication was malignant in 1112 (53%) patients and benign in 993 (47%).

The IONM devices utilized were Medtronic® in 844 (40%) patients, Dr Langer® in 1177 (56%), and Inomed® in 84 (4%).

The total procedures included 1258 (60%) total thyroidectomies, 366 (17%) hemithyroidectomies, 240 (12%) total thyroidectomies with central lymph-node dissection (LND), 149 (7%) total thyroidectomies with central and lateral LND, 51 (2,4%) completion thyroidectomies, 36 (1.7%) hemithyroidectomies with central LND, and 5 (0.2%) recurrent operations (revision on the same side). Table 1 shows patient demographics.

**Table 1:** Demographics

	*n* (%)
Gender Male Female Total	479 (23) 1626 (77) 2105
Indication Malignancy Benign	1112 (53) 993 (47)
Nerves at risk	3772
Initial vagus amp. ≥ 500 µV	2896 (77)
IONM device Medtronic® Dr. Langer® Inomed®	844 (40) 1177 (56) 84 (4)
Type of surgery Total thyroidectomy Total thyroidectomy + Central LND Total thyroidectomy + Central LND + Lateral LND Hemithyroidectomy Hemithyroidectomy(+ Central LND) Recurrent Op. (revision same side) Completion thyroidectomy	1258(60) 240(12) 149(7) 366(17) 36(1,7) 5(0,2) 51(2,4)

### IONM outcomes

In this study, the mean initial vagus nerve amplitude was 1093.74 µV (± 861.39). A total of 2896 nerves (77%) had a mean baseline vagus nerve amplitude of 500 µV or higher. IONM amplitudes and latency values are shown in Table 2*.*

**Table 2:** Amplitude and latency values

	Stimulation points	Mean amplitude (µV) (Std)	Mean latency (ms) (Std)
Left	V1	1048.3663(± 836.52906)	6.5249(± 1.282423)
	R1	1302.0713(± 987.24662)	2.2909(± 0.500816)
	R2	1355.4028(± 1050.71900)	2.1986(± 1.380382)
	V2	991.0314(± 837.27008)	6.5960(± 0.555148)
Right	V1	1139.1137(± 886.25094)	4.8306(± 0.91860)
	R1	1365.6225(± 1001.29966)	2.2823(± 0.65022)
	R2	1449.1878(± 1066.62968)	2.1427(± 0.96937)
	V2	1138.9612(± 923.00131)	4.7700(± 0.583142)

### Surgical outcomes

Among these patients, 99 (6%) out of 2,105 experienced LOS (Type 1 or Type 2) or AE during IONM. Of these 99 patients, 53 (2.5%) had Type 1 LOS, 36 (2%) had AE, and 10 (0.5%) had Type 2 LOS. The mechanisms of injury observed in these 99 patients were as follows: 45 (45%) unclear, 33 (33%) traction, 9 (9%) mechanical trauma, 7 (8%) transection, and 5 (5%) thermal injury. Table 3 shows the number of patients with LOS and AE, as well as the causes of injury mechanisms.

**Table 3:** LOS and AE & ınjury mechanism

	*n* (%)
LOS and AE LOS type 1 LOS type 2 AE None	53 (2.5) 10(0.5) 36 (2) 2006 (95)
Injury mechanism Unclear Traction Mechanical Trauma Transection Thermal	45 (42) 33 (33) 9 (9) 7 (8) 5 (5)

In patients with Type 1 LOS, the mechanisms of injury were 23 (43%) traction, 11 (21%) unclear, 8 (15%) mechanical trauma, 7 (13%) transection, and 4 (8%) thermal injury, listed in order of frequency. In 10 patients with Type 2 LOS, the cause of the injury mechanism was observed to be unclear. In patients with AE, the mechanisms of injury were 24 (66%) unclear, 10 (28%) traction, 1 (3%) mechanical trauma, and 1 (3%) thermal injury, listed in order of frequency. Visualizing the RLN anatomically during thyroid surgery does not necessarily guarantee the prevention of nerve injury. Observed RLN injuries and corresponding IONM responses, such as LOS and AE, are also understood to reflect underlying injury mechanisms and the severity of the damage. These findings were statistically significant (*p* < 0.001). Table 4 presents the types of LOS and AE observed in patients and indicates the corresponding injury mechanisms associated with each case.

**Table 4:** LOS or AE and injury mechanism

	Unclear *N* (%)	Traction *N* (%)	Mechanical trauma *N* (%)	Thermal *N* (%)	Transection *N* (%)	Total *N* (%)	
LOS type 1	11 (21)	23 (43)	8 (15)	4 (8)	7 (13)	53 (100)	
LOS type 2	10 (100)	0	0	0	0	10 (100)	
AE	24 (66)	10 (28)	1 (3)	1 (3)	0	36 (100)	
Total	45 (42)	33 (33)	9 (9)	5 (5)	7 (8)	99 (100)	
						*p < 0.001	

Among the 99 patients with LOS or AE, 48 (49%) were classified as having no recovery, 33 (33%) were classified as Yes/No (< 50% recovery or R1), and 18 (18%) were classified as Yes (> 50% recovery or R1) in the evaluation of intra-operative recovery of LOS or AE.

Regarding the relationship between the mechanism of injury and intra-operative recovery of LOS or AE, the mechanisms in patients with no recovery (No) were as follows: 15 (31%) unclear, 13 (27%) traction, 8 (17%) mechanical trauma, 7 (14.5%) transection, and 5 (10.5%) thermal injury, in order of frequency. In patients with Yes/No (< 50% recovery or R1), the mechanisms were 17 (51%) unclear and 16 (49%) traction. In patients with Yes (> 50% recovery or R1), the mechanisms were 13 (72%) unclear, 4 (22%) traction, and 1 (6%) mechanical trauma (*p* < 0.001). Table 5 illustrates the intra-operative recovery response observed in cases of LOS or AE caused by an injury mechanism.

**Table 5:** Injury mechanism and ıntraoperative recovery of LOS or AE

	No *n* (%)	Yes/No (< %50 Rd or R1) *n* (%)	Yes (> %50 Rd or R1) *n* (%)	Total *n* (%)
Unclear	15 (33)	17 (38)	13 (29)	45 (100)
Traction	13 (39)	16 (49)	4 (12)	33 (100)
Mechanical trauma	8(89)	0	1 (11)	9 (100)
Thermal	5 (100)	0	0	5 (100)
Transection	7 (100)	0	0	7 (100)
Total	48 (49)	33 (33)	18 (18)	99 (100) *p < 0.001

Intraoperative recovery of LOS or AE in relation to the mechanism of trauma, the most common situation is unclear and this situation is thought to be most likely due to the other additional common cause, traction. We see partial or complete recovery especially in these two causes, but it is also seen that the signal loss is irreversible as the severity of the damage increases (transection, mechanical trauma, and thermal injury).

In the post-operative day 1 vocal cord examination performed in the 99 patients with LOS or AE, post-operative VCP was observed in 46 (87%) of the 53 patients with LOS Type 1, 9 (90%) of the 10 patients with LOS Type 2, and 2 (6%) of the 36 patients with AE, resulting in a total of 57 patients (2.7% of the 2,105 patients), with statistical significance (*p* < 0.001). Table 6 shows whether VCP was observed in vocal cord examinations on post-operative day 1 in patients with documented LOS or AE.

**Table 6:** LOS, AE, and VCP in the PO day 1

	Yes *n* (%)	No *n* (%)	Total *n* (%)
LOS type 1	46 (87)	7 (13)	53 (100)
LOS type 2	9 (90)	1 (10)	10 (100)
AE	2 (6)	34 (94)	36 (100)
Total	57 (59)	42 (41)	99 (100) *p < 0.001

These 57 patients with post-operative VCP were further evaluated for permanent VCP. Among the 46 patients with LOS Type 1, 8 (17%) had permanent VCP, and 1 (11%) of the 9 patients with LOS Type 2 had permanent VCP, resulting in a total of 9 patients (0.4% of the 2,105 patients) with permanent VCP. No permanent VCP was observed in patients with AE, with statistical significance (*p* = 0.016). Table 7 shows whether permanent VCP developed during long-term follow-up in patients with LOS or AE and vocal cord paralysis on PO day 1.

**Table 7:** LOS, AE, and permanent VCP

	Yes *n* (%)	No *n* (%)	Total *n* (%)
LOS type 1	8 (17)	38 (83)	46 (100)
LOS type 2	1 (11)	8 (89)	9 (100)
AE	0	2 (100)	2 (100)
Total	9 (16)	48 (84)	57 (100) *p = 0.016

In a standard IONM defined by INMSG, the definitions of LOS Type 1, LOS Type 2, and AE are also provided in the measurements section. These intra-operative definitions carry predictive value regarding the occurrence of VCP. This predictive capability allows the surgeon to adjust the surgical technique accordingly and, if necessary, consider a staged surgical plan based on the intra-operative findings.

Such an approach guides the surgeon toward preventing RLN injury or, if an injury occurs, minimizing the severity of the damage. Consequently, this strategy not only contributes to predicting VCP on day 1 but also helps prevent permanent VCP if VCP is anticipated. Furthermore, this approach has been shown to be statistically significant in predicting both day 1 VCP and permanent VCP.

Finally, the occurrence of LOS and AE, injury mechanisms, PO Day 1 VCP, and permanent VCP in each center has also been specified in the tables. These findings are shown in Tables 8, 9, 10, and 11*.*

**Table 8:** Countries and LOS or AE

	LOS Type 1 *n* (%)	LOS Type 2 *n* (%)	AE *n* (%)	Total *n* (%)
Turkey	10(35)	5(16)	14(49)	29(100)
Taiwan	19(73)	0	7(27)	26(100)
Malaysia	24(55)	5(11)	15(34)	44(100)
France	0	0	0	0
Serbia	0	0	0	0
Total	53(54)	10(10)	36(36)	99(100)

**Table 9:** Countries and ınjury mechanisms

	Unclear *n* (%)	Traction *n* (%)	Mechanical trauma *n* (%)	Thermal *n* (%)	Transection *n* (%)	Total *n* (%)
Turkey	17(63)	9(33)	0	0	1(4)	27(100)
Taiwan	5(19)	16(61)	3(12)	2(8)	0	26(100)
Malaysia	23(50)	8(17)	6(13)	3(7)	6(13)	46(100)
France	0	0	0	0	0	0
Serbia	0	0	0	0	0	0
Total	45(46)	33(33)	9(9)	5(5)	7(7)	99(100)

**Table 10:** Countries and PO Day1 VCP

	Yes *n* (%)	No *n* (%)	Total *n* (%)
Turkey	12(44)	15(56)	27(100)
Taiwan	17(65)	9(35)	26(100)
Malaysia	28(61)	18(39)	46(100)
France	0	0	0
Serbia	0	0	0
Total	57(58)	42(42)	99(100)

**Table 11:** Countries and permanent VCP

	Yes *n* (%)	No *n* (%)	Total *n* (%)
Turkey	1(8)	11(92)	12(100)
Taiwan	2(12)	15(88)	17(100)
Malaysia	6(21)	22(79)	28(100)
France	0	0	0
Serbia	0	0	0
Total	9(16)	48(84)	57(100)

In our study, the use of TCN electrodes did not result in any needle-related complications, such as bleeding, intracartilaginous hematoma, endotracheal tube cuff eruption, or needle breakage.

## Discussion

An initial amplitude of 500 µV or higher is an achievable and reliable EMG target [1]. In this study, a mean initial amplitude of 1093.74 µV was found. In addition to the comparison between ETT electrodes and TCN electrodes, several other techniques have also been compared with ETT electrodes. In many studies using electrodes that are sutured to the thyroid cartilage for IONM, TCN electrodes, subdermal needle electrodes applied to the subperichondrium of the lateral thyroid cartilage, and fully percutaneous needle electrodes made using the porcine model, it has been determined that initial amplitude values are higher than those of ETT electrodes [2–14]. Higher initial EMG amplitudes assist in the localization and identification of the nerve during RLN monitoring [14]. Higher initial amplitudes also facilitate the detection of anatomical variations in RLN, aiding in the prevention of nerve injury in patients with such variations [15].

In a study comparing EMG signals during IONM using both needle electrodes applied to the subperichondrium of the lateral thyroid cartilage and ETT electrodes, it was observed that needle electrodes provided more stable EMG values throughout the procedure. Additionally, the higher initial EMG amplitudes obtained with needle electrodes were found to be helpful in providing an early warning of adverse EMG changes caused by RLN traction stress [8].

Ultimately, higher EMG amplitudes increase the sensitivity of RLN monitoring, allowing surgeons to more effectively prevent RLN injury [12, 16, 17]. However, in the study conducted by Slycke et al., where electrodes were sutured to the thyroid cartilage, and in the research by Wu et al. involving transcutaneous recording, initial amplitudes were observed to be lower compared to TCN electrodes. Huang et al. described the use of full percutaneous electrodes as an alternative method, which, like other methods, cannot be used continuously. Furthermore, this approach is not universally applicable to all patients, as there is limited clinical experience, and IONM instruments can restrict the surgical field. If not carefully applied, there is a risk of cricothyroid muscle or EBSLN injury in cases of overly caudal insertion [2, 4, 13].

For a high-quality IONM recording with ETT electrodes, maintaining contact between the electrodes and the vocal cords is essential. However, if the size of the ETT used to ensure proper contact is too large, replacing it with a smaller ETT electrode may result in inadequate contact [1].

False loss of signal (LOS) can occur due to insufficient contact with ETT electrodes. As a result, verifying the position of ETT electrodes and ensuring proper repositioning can lead to time delays. Surgical manipulations during the procedure can affect the amplitude and latency values obtained. Compared to TCN electrodes, this method is more expensive. TCN electrodes, however, do not require the numerous prerequisites and intra-operative adjustments needed for high-quality recordings in IONM performed with ETT electrodes.

The use of ETT electrodes is the most widely used recording method. However, TCN electrodes are already included in guidelines [18]. Many studies with smaller patient populations and experimental studies have found that TCN electrodes have several advantages over ETT electrodes [3, 8, 9, 19]. Additionally, several studies have shown that TCN electrodes are more cost-effective, being approximately 20–30 times cheaper compared to ETT electrodes [5, 6].

The advantages of TCN electrodes include ease of use, control by the surgeon, and feasibility, as the surgeon can reinsert or remove the needle easily in case of needle displacement or after the operation. They are also safe, as they are placed in the avascular plane of the thyroid cartilage, and can be used in patients with tracheostomy [6, 7].

TCN electrodes appear to have predictive value for the risk of post-operative vocal cord paralysis or long-term permanent vocal cord paralysis by detecting in real time a nerve that has developed LOS or AE, is developing LOS or AE or has reversed LOS or AE. The absence of intra-operative false-positive LOS and this predictive value strengthens the surgeon’s decision-making regarding whether to perform staged surgery. In this study, no false LOS was observed, which is consistent with other studies using TCN electrodes [6, 8].

However, our study has limitations. Three different types of devices from different brands were used. Although the patients were operated on by experienced surgeons, the surgeries were performed at different centers. There were missing data and limitations in data quality measures. The scale of the clinics was non-homogeneous, and no data on EBSLN were available. It is unclear which patients received continuous versus intermittent IONM or whether monopolar or bipolar stimulator probes were used.

TCN electrodes are invasive, require additional dissection, and carry a risk of hematoma. There is also a risk of endotracheal cuff rupture or needle breakage, although no needle-related complications occurred in this study. In particular, there is always a risk of needlestick injury with needle electrodes during IONM. TCN electrodes are difficult to use in certain cases, such as with giant goiter or calcified cartilage. If the thyroid cartilage is calcified, it is more convenient to place the needle electrodes in the subperichondrium or cricothyroid membrane instead of the cartilage. Another limitation of the TCN electrode is that it cannot be used during endoscopic or remote access thyroidectomy, especially in the transoral endoscopic approach.

Each centers consistently use TCN electrodes, and their application is neither contraindicated nor impossible in cases of giant goiter and/or calcified thyroid cartilage. However, these conditions may affect user comfort, necessitating adjustments and re-evaluations of needle positioning. For this reason, surgeons may opt to place the electrodes in the subperichondrium or cricothyroid membrane. In our study, particularly in cases involving giant goiter and/or calcified thyroid cartilage, positioning the needle electrodes in the subperichondrium or cricothyroid membrane was considered a viable alternative. Our findings indicate that the accuracy of IONM data remains unchanged regardless of the placement technique. However, subperichondrial placement carries a risk of needle displacement and breakage, while placement in the cricothyroid membrane presents a potential risk of EBSLN injury.

If this technique is used during the initial surgery of a patient who has previously undergone thyroid surgery, the completion of pyramidal lobe dissection and the exposure of the thyroid cartilage lamina inherently ensure that the same technique can be reapplied in any future surgical procedures. In our study, revision and completion thyroid surgeries were included, further demonstrating that this technique can be effectively utilized in repeat procedures.

The low cost of TCN electrodes, along with their easy replacement and reapplication in rare cases of technical issues such as needle displacement or breakage during surgery, makes them a more practical alternative to ETT electrodes.

The mention of system manufacturers in our study is not intended to compare different devices. Instead, it aims to highlight that the use of TCN electrodes is not restricted to a specific brand, emphasizing their ease of application and ensuring that IONM data outcomes are not dependent on a particular manufacturer. Notably, no brand-related complications were observed in any of the participating centers.

Due to various limitations associated with ETT electrodes, such as false-positive LOS from endotracheal tube malposition, tube rotation, saliva accumulation, lack of direct surgeon control, time-consuming repositioning, risk of inadequate contact to vocal cords, and high cost, TCN electrodes are preferred. In each center, the IONM technique utilizing TCN electrodes is routinely employed.

The centers in France and Turkey completely use needle electrodes, while the center in Taiwan utilizes them in more than 80% of cases. The center in Malaysia employs needle electrodes in approximately 40% of cases, whereas the center in Serbia has recently started using them routinely and applies them in around 20% of cases. Overall, the reason for the lower usage compared to the conventional method is that this technique has only recently gained popularity.

The use of TCN electrodes initially emerged as an alternative due to the various disadvantages associated with ETT electrodes. It was first tested in porcine models and later evaluated in multiple studies comparing TCN and ETT electrodes simultaneously. Given that RLN injury is the most concerning complication in thyroid surgery, several studies have reported favorable outcomes for TCN electrodes in preventing this risk.

However, the adoption of TCN electrodes has not yet become widespread compared to ETT electrodes. This is mainly due to the smaller sample sizes in studies focusing on TCN electrodes, the larger number of studies conducted with ETT electrodes, and the broader clinical experience with ETT electrodes.

In our study, each participating center employed IONM with TCN electrodes. This decision was based on the presence of numerous prior studies demonstrating the effectiveness and significance of TCN electrodes, as well as the fact that the disadvantages observed with the ETT electrode method were not encountered when using TCN electrodes in IONM.

Our study includes data from patients who underwent surgery between January 2018 and August 2023, encompassing the period of the COVID-19 pandemic, which affected surgical practices worldwide to varying degrees. However, as indicated in a systematic review on the feasibility of thyroid surgery during the COVID-19 pandemic, our study also demonstrates that the pandemic did not impact the applicability of thyroid surgery [20].

## Conclusion

TCN electrodes have proven effective in monitoring nerve activity, with high sensitivity for detecting potential nerve injuries. The low incidence of VCP indicates the high predictive accuracy of TCN electrodes. TCN electrodes offer a cost-effective, easy-to-use, sensitive, and highly reliable method. It is recommended to standardize TCN electrodes as an alternative recording-side method to ETT electrodes during IONM.

## Data Availability

The data that support the findings of this study are available from the corresponding author upon reasonable request.
